# Supplementary material for: Heat adaptation of phage T7 under an extended genetic code
Source: Virus Evol. 2021 Dec 1;7(2):veab100. doi: 10.1093/ve/veab100 (PMC8923235; doi:10.1093/ve/veab100)
Supplement: veab100_Supp [file veab100_supp.zip › Sequencing_Analysis.zip!/Sequencing_Analysis/A2/evidence/JC_SIDE_2_56.html]

BRESEQ :: Evidence


| New junction evidence | | | | | | | | | | | |
| --- | --- | --- | --- | --- | --- | --- | --- | --- | --- | --- | --- |
|  | | seq id | position | reads (cov) | reads (cov) | score | skew | freq | annotation | gene | product |
| \* | ? | NC\_001604.1 | = 27753 | 287 (0.670) | 183 (0.420) +CGCTATATCTGG | 105/544 | 1.5 | 38.0% | intergenic (–/–) | *–/–* | –/– |
|  | ? | NC\_001604.1 | 27754 = | 318 (0.700) |  |  |  |  | intergenic (–/–) | *–/–* | –/– |

Only 100 of 498 total aligned reads displayed.  

`ATCGGTGGTAACTGCGGGGACCAGTGCTGGTTCGTTACGAGCGACCAAGTGTGGCGACTTAGTGGAAAGGCTAAGCGAAAGTTCCGTAAGTTAATCATGGAGTATCGCGATAAGATGCTTGAGAAGTATGATACTCTTTGGAATTACGTATGGGTAGGCAATACGTCCCACATTCGTTTCCTCAAGACTATCGGTGCGGTATTCCATGAAGAGTACACACGAGATGGTCAATTTCAGTTATTTACAATCACGAAAGGAGGATAACCATATGTGTTGGGCAGCCGCAATACCTATCGCTATATCTGGCGCTCAGGCTATCAGTGGTCAGAACGCTCAGGCCAAAATGATTGCCGCTCAGACCGCTGCTGGTCGTCGTCAAGCTATGGAAATCATGAGGCAGACGAACATCCAGAATGCTGACCTATCGTTGCAAGCTCGAAGTAAACTTGAGGAAGCGTCCGCCGAGTTGACCTCACAGAACATGCAGAAGGTCCAAGCTATTGGGTCTATCCGAGCGGCTATCGGAGAGAGTATGCTTGAAGGTTCCTCAATGGACCGCATTAAGCGAGTCACAGAAGGACAGTTCATTCGGGAA  >  NC_001604.1/27460‑28054`  

                                                                                                                                                                                                                                                                                                      `|`                                                                                                                                                                                                                                                                                                              
`ttaagatccaggtgctggttccttatcagtgcacaagtgtggctacttatttgaaaggcgaagagaaagtaccgtaagttaatccttgagtatcgcgataagatgcttaataactattaaacactttggaattacgtattggtaggcaatacgtcccacattcgtttcctcaatactatcggtgcagtattccatgaagagttcacacgagatgttcaatttcagttatttacaatcacgaaaggaggataaccatatgtgttgggcagccgcaatacctatcgctatatctggcactata                                                                                                                                                                                                                                                                                                        <  1:100160‑M2/7‑1 (MQ=255)`  
                  `gACCAGTGCTGGTTCGTTACGAGCGACCAAGTGTGGCGACTTAGTGGAAAGGCTAAGCGAAAGTTCCGTAAGTTAATCATGGAGTATCGCGATAAGATGCTTGAGAAGTATGATACTCTTTGGAATTACGTATGGGTAGGCAATACGTCCCACATTCGTTTCCTCAAGACTATCGGTGCGGTATTCCATGAAGAGTACACACGAGATGGTCAATTTCAGTTATTTACAATCACGAAAGGAGGATAACCATATGTGTTGGGCAGCCGCAATAACTATCGCTATATCTGGCGCTCAGGCTATc                                                                                                                                                                                                                                                                                      >  1:779669/1‑301 (MQ=255)`  
                    `ggggctcggagatgtgtataagagacaggtgtGGCGACTTAGTGGAAAGGCTAAGCGAAAGTTCCGTAAGTTAATCATGGAGTATCGCGATAAGATGCTTGAGAAGTATGATACTCTTTGGAATTACGTATGGGTAGGCAATACGTCCCACATTCGTTTCCTCAAGACTATCGGTGTGGTATTCCATGAAGAGTACACACGAGATGGTCAATTTCAGTTATTTACAATCACGAAAGGAGGATAACCATATGTGTTGGGCAGCCGCAATACCTATCGCTATATCTGGCGCTCAGGCTATCAg                                                                                                                                                                                                                                                                                    <  1:497051/273‑1 (MQ=255)`  
                    `aagagacaggTTCGTTACGAGCGACCAAGTGTGGCGACTTAGTGGAAAGGCTAAGCGAAAGTTCCGTAAGTTAATCATGGAGTATCGCGATAAGATGCTTGAGAAGTATGATACTCTTTGGAATTACGTATGGGTAGGCAATACGTCACACATTCGTTTCCTCAAGACTATCGGTGCGGTATTCCATGAAGAGTACACACGAGATGGTCAATTTCAGTTATTTACAATCACGAAAGGAGGATAACCATATGTGTTGGGCAGCCGCAATACCTATCGCTATATCTGGCGCTCAGGCTATCAg                                                                                                                                                                                                                                                                                    <  1:636350/293‑1 (MQ=255)`  
                    `aaaGTGCTGGTTCGTTACGAGCGACCAAGTGTGGCGACTTAGTGGAAAGGCTAAGCGAAAGTTCCGTAAGTTAATCATGGAGTATCGCGATAAGATGCTTGAGAAGTATGATACTCTTTGGAATTACGTATGGGTAGGCAATACGTCCCACATTCGTTTCCTCAAGACTATAGGTGCGGTATTCCATGAAGAGTACACACGAGATGGTCAATTTCAGTTATTTACAATCACGAAAGGAGGATAACCATATGTGTTGGGCAGCCGCAATACCTATCGCTATATCTGGCGCTCAGGCTATCAg                                                                                                                                                                                                                                                                                    <  1:746436/299‑1 (MQ=255)`  
                              `gaccaagtgtggcgacttagtggaaaggctaagcgaaagttccgtaagttaatcatggagtatcgcgataagatgcttgagaagtatgatactctttggaattacgtatgggtaggcaatacgtcccacattcgtttcctcaagactatcggtgcggtattccatgaagagtacacacgagatggtcaatttcagttatttacaatcacgaaaggaggataaccatatgtgttgggcagccgcaatacctatcgctatatctggCGCTATATCTGGCGCTCAGGCTATCAGTGGTCAGAAc                                                                                                                                                                                                                                                                          >  1:484610‑M2/265‑301 (MQ=255)`  
                              `gaccaagtgtggcgacttagtggaaaggctaagcgaaagttccataagttaatcatggagtatcgcgataagatgcttgagaagtatgatactctttgtaattacggatgggtaggcaatacatcacacatgcgtttcctcaagactatctgtgcggtattcaatgaagattacacacgagatgatcaattgcagttatttacaatcaagaacaaacaataaccatatgtggtgagcagccgcaatacctatcgctatatctggcgctaaatctggcgcaccagcctgcgatggtaagaag                                                                                                                                                                                                                                                                          >  1:168614‑M2/265‑279 (MQ=255)`  
                                 `tgtgtataagagacaggtggaaaggctaagcgaaagttccgtaagttaatcatgtagtatcgcgataagatgcttgagaagtatgatactctttggaattacgtatgggtaggcaatacgtcccacattcgtttcctcaagactatcggtgcggtattccatgaagagtacacacgagatggtcaatttcagttatttacgatcacgaaaggaggataaccatatgtgttgggcagccgcaatacctatcgctatatctggCGCTATATCTGGCGCTCAGGCTATCAGTGGTCAGAACGCt                                                                                                                                                                                                                                                                       <  1:815652‑M2/40‑1 (MQ=255)`  
                                         `cGACCAAGTGTGGCGACTTAGTGGAAAGGCTAAGCGAAAGTTCCGTAAGTTAATCATGGAGTATCACGATAAGATGCTTGAGAAGTATGATACTCTTTGGAATTACGTATGGGTAGGCAATACGTCCCACATTCGTTTCCTCAAGACTATCGGTGCGGTATTCCATGAAGAGTACACATGAGATGGTCAATTTCAGTTATTTACAATCACGAAAGGAGGATAACCATATGTGTTGGGCAGCCGCAATACCTATCGCTATATCTGGCGCTCAGGCTATCAGTGATCAGAACGCTCAGGCCaa                                                                                                                                                                                                                                                               >  1:386559/1‑301 (MQ=255)`  
                                            `atgtgtataagagacaggTGGAAAGGCTAAGCGAAAGTTCCGTAAGTTAATCATGGAGTATCGCGATAAGATGCTTGAGAAGTATGATACTCTTTGGAATTACGTAGGTGTAGGCAATACGTCCCACATTCGTTTCCTCAAGACTATCGGTGCGGTATTCCATGAAGAGTACACACGAGATGGTCAATTTCAGTTATTTACAATCACGAAAGGAGGATAACCATATGTGTTGGGCAGCCGCAATACCTATCGCTATATCTGGCGCTCAGGCTATCAGTGATCAGAACGCTCAGGCCAAAAt                                                                                                                                                                                                                                                            <  1:372244/284‑1 (MQ=255)`  
                                            `agagacagggCGACTTAGTGGAAAGGCTAAGCGAAAGTTCCGTAAGTTAATCATGGAGTATCGCGATAAGATGCTTGAGAAGTATGATACTCTTTGGAATTACGTATGGGTAGGCAATACGTCCAACATTCGTTTCCTCAAGACTATCGGTGCGGTATTCCATGAAGAGTACACACGAGATGGTCAATTTCAGTTATTTACAATCACGAAAGGAGGATAACCATATGTGTTGGGCAGCCGCAATACCTATCGCTATATCTGGCGCTCAGGCTATAAGTGATCAGAACGCTCAGGCCAAAAt                                                                                                                                                                                                                                                            <  1:179142/293‑1 (MQ=255)`  
                                            `acttagtggaaaggctaagcgaaagttccgtaagttaatcatggagtatcgcgataagatgcttgagaagtatgatactctttggaattacgtatgggtaggcaatacgtcccacattcgtttcctcaagactatcggtgcggtattccatgaagagtacacacgagatggtcaatttcagttatttacaatcacgaaaggaggataaccatatgtgttgggcagccgcaatacctatcgctatatctggCGCTATATCTGGCGCTCAGGCTATCAGTGGTCAGAACGCTCAGGccaaatg                                                                                                                                                                                                                                                            <  1:291142‑M2/51‑3 (MQ=255)`  
                                                             `gTGGAAAGGCTAAGCGAAAGTTCCGTAAGTTAATCATGGAGTATCGCGATAAGATGCTTGAGAAGTATGATACTCTTTGGAATTACGTATGGGTAGGCAATACGTCCCACATTCGTTTCCTCAAGACTATCGGTGCGGTATTCCATGAAGAGTACACACGAGATGGTCAATTTCAGTTATTTACAATCACGAAAGGAGGATAACCATATGTGTTGGGCAGCCGCAATACCTATCGCTATATCTGGCGATCAGGCTATCAGTGATCAGAACGCTCAGGCCAACATGAtctgtctcttataca                                                                                                                                                                                                                                           >  1:673719/1‑287 (MQ=255)`  
                                                                 `agagacagaGCGAAAGTTCCGTAAGTTAATCATGGAGTATCGCGATAAGATGCTTGAGAAGTATGATACTCTTTGGAATTACGTATGGGTAGGCAATACGTCCCACATTCGTTTCCTCAAGACTATCGGTGCGGTATTCCATGAAGAGTACACACGAGATGGTCAATTTCAGTTATTTACAATCACTAAAGGAGGATAACCATATGTGTTGGGCAGCCGCAATACCTATCGCTATATCTGGCGCTCAGGCTATCAGTGATCAGAACGCTCAGGCCAAAATGATTGCCGCTCAGACCgctgc                                                                                                                                                                                                                                       <  1:549937/293‑1 (MQ=255)`  
                                                                 `aaagttccgtaagttaatcatggagtatcgcgataagatgcttgagaagtatgatactctttggaattacgtatgggtaggcaatacgtcccacattcgtttcctcaagactatcggtgcggtattccatgaagagtacacacgagatggtcaatttcagttatttacaatcacgaaaggaggataaccatatgtgttgggcagccgcaatacctatcgctatatctggCGCTATATCTGGCGCTCAGGCTATCAGTGGTCGGAACGCTCAGGCCAAAATGATTGCCGCTCAGACCgctgc                                                                                                                                                                                                                                       <  1:187233‑M2/72‑1 (MQ=255)`  
                                                                    `ggCTAAGCGAAAGTTCCGTAAGTTAATCATGGAGTATCGCGATAAGATGCTTGGGAAGTATGATACTCTTTGGAATTACGTATGGGTAGGCAATACGTCCCACATTCGTTTCCTCAAGACTATCGGTGCGGTATTCCATGAAGAGTACACACGAGATGGTCAATTTCAGTTATTTACAATCACGAAAGGAGGATAACCATATGTGTTGGGCAGCCGCAATACCTATCGCTATATCTGGAGCTCAGGCTATCAGTTATCAGAACGCTCAGGCCAAAATGATTGCCTCCCAGCCCGCTGCTgg                                                                                                                                                                                                                                    >  1:418875/1‑301 (MQ=255)`  
                                                                    `ggCTAAGCGAAAGTTCCGTAAGTTAATCATGGAGTATCGCGATAAGATGCTTGAGAAGTATGATACTCTTTGGAATTACGTATGGGTAGGCAATACGTCCCACATTCGTTTCCTCAAGACTATCGGTGCGGTATTCCATGAAGAGTACACACGAGATGGTCAATTTCAGTTATTTACAATCACGAAAGGAGGATAACCATATGTGTTTGGCAGCCGCAATACCTATCGCTATATCTGGCGCTCAGGCTATCAGTGATCAGAACGCTCAGGCCAAAATGATTGCCGCTCAGACCGCTGCTgg                                                                                                                                                                                                                                    >  1:761368/1‑301 (MQ=255)`  
                                                                                `gtataagagacagaTCATGGAGTATCGCGATAAGATGCTTGATAAGTATGATACTTTTTGGAATTACGTATGGGTAGGAAATACGTCCCACATGCGTTTCCTCAAGACTATCGGTGCGGTATTCCATGAAGAGTACACACGAGATGGTCAATTTCAGTTATTTACAATCACGAAAGGAGGATAACCATATGTGTTGGGCAGCCGCAATACCTATCGCTATATCTTGCGCTCAGGCTATCAGTGATCAGAACGCTCAGGCCAAAATGATTGCCGCTCAGACCGCTGCTGGTCGTCGTCAAGc                                                                                                                                                                                                                        <  1:566408/288‑1 (MQ=255)`  
                                                                                       `gagtatcgcgataagatgcttgagaagtatgatactctttgtaattacgtatgggtaggcaatacgtcccacattcgtttcctcaagactatcggtgcggtattccatgaagagtacacacgagatggtcaatttcagttatttacaatcacgaaaggaggataaccatatgtgttgggcagccgcaatacctatcgctatatctggCGCTATATCTGGCGCTCAGGCTATCAGTGGTCATAACGCTCAGGCCAAAATGATTGCCGCTCAGACCGCTGCTGGTCGTCGTCAAGCTATGGaa                                                                                                                                                                                                                 <  1:451915‑M2/94‑1 (MQ=255)`  
                                                                                       `gagtatcgcgataagatgcttgagaagtatgatactctttggaattacgtatgggtaggcaatacgtcccacattcgtttcctcaagactatcggtgcggtattccatgaagagtacacacgagatggtcaatttcagttatttacaatcacgaaaggagaataaccatatgtgttgggcagccgcaatacctatcgctatatctggCGCTATATCTGGCGCTCAGGCTATCAGTGGTCAGAACGCTCAGGCCAAAATGATTGCCGCTCAGACCGCTGCTGGTCGTCGTCAAGCTATGGaa                                                                                                                                                                                                                 <  1:242502‑M2/94‑1 (MQ=255)`  
                                                                                       `aaGTAAATCATGGAGTATCGCGATAAGATGCTTGAGAAGGATGAGACGCTGTGGAATTACGTATGGGTAGGCAATACGTCCCACATTCGTTTCCTCAAGACGAACGGTGCGGTAGTCCATGAAGAGTACACACGAGATGGTCAATTTCAGTTATTGACAATCACGAAAGGAGGATAACCATATGTGGTGAGCAGCCGCAATACCTATCGCTATATCTGGCGCTCAGGCTATCAGTGATCAGAACGCTCAGGCCAAAATGATTGCCGCTCAGACCGCTGCTGGTCGTCGTCAAGCTATGGaa                                                                                                                                                                                                                 <  1:88092/301‑1 (MQ=255)`  
                                                                                         `gtatcgcgataagatgcttgagaagtatgatactctttggaattacgtatgggtaggcaatacgtcccacattcgtttcctcaagactatcggtgcggtattccatgaagagtacacacgagatggtcaatttcagttatttacaatcacgaaaggaggataaccatatgtgttgggcagccgcaatacctatcgctatatctggCGCTATATCTGGCGCTCAGGCTATCAGTGGTCAGAACGCTCAGGCCAAAATGATTGCCGCTCAGACCGCTGCTGGTCGTCGTCAAGCTATGGAAAt                                                                                                                                                                                                               >  1:361507‑M2/206‑301 (MQ=255)`  
                                                                                             `aTCATGGAGTATCGCGATAAGATGCTTGAGAAGTATGATACTCTTTGGAATTACGTATGGGTAGGCAATACGTCCCACATTCGTTTCCTCAAGACTATCGGTGCGGTATTCAATGAAGAGTACACACGAGATGGTCAATTTCAGTTATTTACAATCACGAAAGGAGGATAACCATATGTGTTGGGCAGCCGCAATACCTATCGCTATATCTGGCGCTCAGGCTATCAGTGATCAGAACGCTCAGGCCAAAATGATTGCCGCTCAGACCGCTGCTGGTCGTCGTCAAGCTATGGAAATCATg                                                                                                                                                                                                           >  1:381909/1‑301 (MQ=255)`  
                                                                                                `ctGGAGTATCGCGATAAGATGCTTGAGACGTATGATACTCTTTTGAATTACGTATTGGTAGGCAATACGTCCCACATTCGTTTCCTCAAGACTATCGGTGCTGTATTCCATGAAGAGTACCCACGCGATGGTCAATTTCAGTTATTTACAATCACGAAAGGAGGATAACCATATGTGTTGGGCAGCCGCAATACCTATCGCTATATCTGGCGCTCAGGCTATCAGTTATCAGAACGCTCAGGCCAAAATGATTGCCGCTCAGACCGCTGCTGGTCGTCGTCAAGCTATGGAAATCATGAgg                                                                                                                                                                                                        <  1:785364/300‑1 (MQ=255)`  
                                                                                                          `gcgATAAGATGCTTGAGAAGTATGATACTCTTTGGAATTACGTATGGGTAGGCAATACGTCCCACATTCGTTTCCTCAAGACTATCGGTGCGGTATTCCATGAAGAGTACACACGAGATGGTCAATTTCAGTTATTTACAATCACGAAAGGAGGATAACCATATGTGTTGGGCAGCCGCAATACCTATCGCTATATCTGGCGCTCAGGCTATCAGTGGTCAGAACGCTCAGGCCAAAATGATTGCCGCTCAGACCGCTGCTGGTCGTCGTCAAGCTATGGAAATCATGAGGCAGACTAACa                                                                                                                                                                                              >  1:158322/1‑301 (MQ=255)`  
                                                                                                            `gATAAGATGCTTGAGAAGTATGATACTCTTTGGAATTACGTATGGGTAGGCAATACGTCCCACATTCGTTTCATCAAGACTATCGGTGCGGTATTCCATGAAGAGTACACACGAGATGGTCAATTTCAGTTATTTACAATCACGAAAGGAGGATAACCATATGTGTTGGGCAGCCGCAATACCTATCGCTATATCTGGCGCTCAGGCTATCAGTGATCAGAACGCTCAGGCCAAAATGATTGCCGCTCAGACCGCTGCTGGTCGTCGTCAAGCTATGGAAATCATGAGGCAGACGAAAATc                                                                                                                                                                                            <  1:604668/301‑1 (MQ=255)`  
                                                                                                             `ctcggagatgtgtataagagacagacgtatgggtaggcaatacgtcccacattcgtttcctcaagactatcggtgcggtattccatgaagagtacacacgagatggtcaatttcagttatttacaatcacgaaaggaggataaccatatgtgttgggcagccgcaatacctatcgctatatctggCGCTATATCTGGCGCTAAGGCTATCAGTGGTCAGAACGCTCAGGCCAAAATGATTGCCGCTCAGACCGCTGCTGGTCGTCGTCAAGCTATGGAAATCATGAGGCAGACGAACATcc                                                                                                                                                                                           <  1:67763‑M2/116‑1 (MQ=255)`  
                                                                                                             `aTAAGATGCTTGAGAAGTATGATACTCTTTGGAATTACGTATGGGTAGGCAATACGTCCCACATTCGTTTCCTCAAGACTATCGGTGCGGTATTCCATGAAGAGTACACACGAGATGGTCAATTTCAGTTATTTACAATCACGAAAGGAGGATAACCATATGTGTTTGGCAGCCGCAATACCTATCGCTATATCTGGCGCTCAGGCTATCAGTGATCAGAACGCTCAGGCCAAAATGATTGCCGCTCAGACCGCTGCTGGTCGTCGTCAAGCTATGGAAATCATGAGGCAGACGAACATcc                                                                                                                                                                                           >  1:521494/1‑301 (MQ=255)`  
                                                                                                               `gggagatggggataagggaccgctgtttGGAATTACGTAGGGGTAGGCACTACGACCCACATTCGTTTCCTCAAGACTATCGGTGCGGTATTCCATGAAGAGTACACACGAGATGGTCAATTTCAGTTATTTACAATCACGAAAGGAGGATAACCATATGTGTTGGGCAGCCGCAATACCTATCGCTATATCTGGCGCTCAGGCTATCAGTGATCAGAACGCTCAGGCCAAAATGATTGCCGCTCAGACCGCGGCTGGTCGTAGTCAAGCTATGGAAATCATGAGGCAGACGAACATCCAg                                                                                                                                                                                         <  1:797081/276‑1 (MQ=255)`  
                                                                                                                     `cTTGAGAAGTATGATACTCTTTGGAATTACGTATGGGTAGGCAATACGTCCCACATTCGTTTCCTCAAGACTATCGGTGCGGTATTCCATGAAGAGTACACACGAGATGGTCAATTTCAGTTATTTACAATCACGAAAGGAGGATAACCATATGTGTTGGGCAGCCGCAATACCTATCGCTATATCTGGCGCTCAGGCTATCAGTGATCAGAACGCTCAGGCCAAAAGGATGGCCGCTCAGACCGCTGCTGGTCGTCGTCAAGCTATGGaactgtctcttatacacatctccgagcgcacg                                                                                                                                                                                   >  1:436818/1‑271 (MQ=255)`  
                                                                                                                           `ctttggaattacgtatgggtaggcaatacgtcccacatttgtttcctcaagactatcggtgcggtattccatgaagagtacacacgagatggtcaatttcagttatttacaatcacgaaaggaggataaccatatgtgttgggcagccgcaatacctatcgctatatctggCGCTATATCTGGCGCTCAGGCTATCAGTGGTCAGAACGCTCAGGCCAAAATGATTGCCGCTCAGACCGCTGCTGGTCGTCGTCAAGCTCTGGAAATCATGaggcagacgaacatccctgtctcttatccc                                                                                                                                                                             >  1:236588‑M2/172‑287 (MQ=255)`  
                                                                                                                               `aTGATACTCTTTGGAATTACGTATGGGTAGGCAATACGTCCCACATTCGTTTCCTCAAGACTATCGGTGCGGTATTCCATGAAGAGTACACACGAGATGGTCAATTTCAGTTATTTACAATCACGAAAGGAGGATAACCATATGTGTTGGGCAGCCGCAATACCTATCGCTATATCTGGCGCTCAGGCTATCAGTGATCAGAACGCTCAGGACAAAATGATTGCCGCTCAGACCGCTGCTGGTCGTCGTCAAGCTATGGAAATCATGAGGCAGACGCACATCCAGAATGCTGACCTATCgc                                                                                                                                                                         >  1:277630/1‑300 (MQ=255)`  
                                                                                                                                 `gATACTCTTTGGAATTACTTATGGGTAGGCAATACGTCCCACATTCGTTTCCTCAAGCCTATCGGTGCGGTATTCCATGAAGAGTACACACGAGATGGTCAATTTCAGTTATTTACAATCACGAAAGTAGGATAACCATATGTGTTGGGCAGCCGCAATACCTATCGCTATATCTGGCGCTCAGGCTATCAGTGATCAGAACGCTCAGGCCAAAATGATTGCCGCTCAGACCGCTGCTGGTCGTCGTCAAGCTATGGAAATCATGAGGCAGACGAACATCCAGAATGCTGACCTATCGTTg                                                                                                                                                                       <  1:561185/301‑1 (MQ=255)`  
                                                                                                                                   `ttacggatgggtaggcaatacgtcccacattcgtttcctcaagactatcggtgcggtattccatgaagagtacacacgagatggtcaatttcagttatttacaatcacgaaaggaggataaccatatgtgttgggcagccgcaatacctatcgctatatctggTGCTATATCTGGCGCTCAGGCTATCAGTGATCAGAACGCTCAGGCCAAAATGATTGCCGCTCAGACCGCTGCTGGTCGTCGTCAAGCTATGGAAATCATGAGGCAGACGAACATCCAGAATGCTGACCTATCGTTGCa                                                                                                                                                                     <  1:336235‑M2/138‑1 (MQ=255)`  
                                                                                                                                     `acgtatgggtaggcaatacgtcccacattcgtttcctcaagactatcggtgcggtattcaatgaagagtacacacgagatggtcaatttcagttatttacaatcatgaaaggaggataaccatatgtgttgggcagccgcaatacctatcgctatatctggCGCTATATCTGGCGCTCAGGCTATCAGTGATCAGAACGCTCAGGCCAAAATGATTGCCGCTCAGACCGCTGCTGGTCGTCGTCAAGCTATGGAAATCATGAGGCAGACGAACATCCAGAATGCTGACCTATCGTTGCAAg                                                                                                                                                                   >  1:533875‑M2/162‑301 (MQ=255)`  
                                                                                                                                       `ctTTTGAATTACGTATGGGTAGGCAATACGTCCCACATTCGTTTCCTCAAGACTATCGGTGCGGTATTCCATGAAGAGTACACACGAGATGGCCAATCTCAGTTATTTACAATCACGAAAGGAGGATAACCATATGTGTTGGGCAGCTGCAATACCTATCGCTATATTTGGCGCTCAGGCTATCAGTGATCAGAATGCTAAGGCCAAAATGATTGCCGCTCAGACCGCTGCTGGTCGTCGTCAAGCTATGGAAATAACGAGACAGACGAACATCCAGAATGCTGCACTATCGTCGCAAGcc                                                                                                                                                                 >  1:66731/1‑300 (MQ=255)`  
                                                                                                                                            `gAATTACGTATGGGTAGGCAATACGTCCCACATTCGTTTCCTCAAGACTATCGGTGCGGTATTCCATGAAGAGTACACACGAGATGGTCAATTTCAGTTATTTACAATCACGAAAGGAGGATAACCATATGTGTTGGGCAGCCGCAATACCTATCGCTATATCTGGCGCTCAGGCTATCAGTGATCAGAACGCTCAGGCCAAAATGATTGCCGCTCAGACCGCTGCTGGTCGTCGTCAAGCTATGGAAATCATGAGGCAGACGAACATCCAGAATGCTGACCTATCGTTGCAAGCTCGAAg                                                                                                                                                            <  1:60152/301‑1 (MQ=255)`  
                                                                                                                                                  `caatacgtcccacattcgtttcctcaagactatcggtgcggtattccatgaagagtacacacgagatggtcaatttcagttatttacaatcacgaaaggaggataaccatatgtgttgggcagccgcaatacatatcgctatatctggCGCTATATCTGGCGCTCAGGCTATCAGTGGTCAGAACGCTCAGGCCAAAATGATTGCCGCTCAGACCGCTGCTGGTCGTCGTCAAGCTATGGAAATCATGAGGCAGACGAACATCCAGAATGCTGACATATCGTTGCAAGCTCGAAGTAAACt                                                                                                                                                      <  1:657286‑M2/153‑1 (MQ=255)`  
                                                                                                                                                        `ggTAGGCAATACGTCCCACATTCGTTTCCTCAAGACTATCGGTGCGGTATTCCATGAAGAGTACACACGAGATGGTCAATTTCAGTTATTTACAATCACAAAAGGAGGATAACCATATGTGTTGGGCAGCCGCAATACCTATCGCTATATCTGGCGCTCAGGCTATCAGTGATCAGAACGCTCAGGCCAAAATGATTGCAGCTAAGACCGCTGCTGGTCGTCGTCAAGCTATGGAAATCATGAGGCAGACGAACATCCAGAATGCTGACCTATCGTTGCAAGCTCGAAGTAAACTTGAGGa                                                                                                                                                <  1:445911/301‑1 (MQ=255)`  
                                                                                                                                                           `aGGCAATACGTCCCACATTCGTTTCCTCAAGACTATCGGTGCGGTATTCCATGAAGAGTACACACGAGATGGTCAATTTCAGTTATTTACAATCACGAAAGGAGGATAACCATATGTGTTGGGCAGCCGCAATACCTATCGCTATATCTGGCGCTCAGGCTATCAGTGATCAGAACGCTCAGGCCAAAATGATTGCCGCTCAGACCGCTGCTGGTCGTCGTCAAGCTATGGAAATCATGAGGCAGACGAACATCCAGAATGCTGACCTATCGTTGCAAGCTCGAAGTAAACTTGAGGAAGc                                                                                                                                             >  1:303538/1‑301 (MQ=255)`  
                                                                                                                                                                       `cctcaagactatcggtgcggtattccattaagagtacacacgagattgtcaatttcagttatttacaatcacgaaaggaggataaccatatgtgtttggcagccgcaatacctatcgctatatctggCGCTATATCTGGCGCTCAGGCTATCAGTGATCAGAACGCTCAGGCCAAAATGATTGCCGCTCAGACCGCTGCTGGTCGTCGTCAAGCTATGGAAATCATGAGGCAGACGAACATCCAGAATGCTGACCTATCGTTGCAAGCTCGAAGTAAACTTGAGTAACCGTCCGCCgagct                                                                                                                                 >  1:479406‑M2/128‑299 (MQ=255)`  
                                                                                                                                                                           `aagactatctgtgcggtattccatgaagagtacacacgagatggtcaatttcagttatttacaatcacgaaaggaggataaccatatgtgttgggcagccgcaatacctatcgctatatctggCGCTATATCGTGCGCTCAGGCTATCAGTGGTCAGAACGCTCAGGCCAAAATGATTGCCGCTCAGACTGCTGCTGGTCGTCGTCAAGCTATGGAAATCATGAGGCAGACGAACATCCAGAATGCTGACCTATCGTTGCAAGCTCGAAGTAAACTTGAGGAAGCGTCCGCCGAGTTGAcc                                                                                                                             <  1:24854‑M2/178‑1 (MQ=255)`  
                                                                                                                                                                                 `tcggagatgtgtataagagacagaTTCCATGAAGAGTACACACGAGATGGTCAATTTCAGTTATTTACAATCACGAAAGGAGGATAACCATATGTGTTGGGCAGCCGCAATACCTATCGCTATATCTGGCGCTCAGGCTATCAGTGATCAGAACGCTCAGGCCAAAATGATTGCCGCTCAGACCGCTGCTGGTCGTCGTCAAGCTATGGAAATCATGAGGCAGACGAACATCCAGAATGCTGACCTATCGTTGCAAGCTCGAAGTAAACTTGAGGAAGCGTCCGCCGAGTTGACATCACAg                                                                                                                       <  1:350219/278‑1 (MQ=255)`  
                                                                                                                                                                                    `taagagacagtccatgaagagtacacacgagatggtcaatttcagttatttacaatcacgaaaggaggataaccatatgtgttgggcagccgcaatacctatcgctatatctggCGCTATATCTGGCGCTCAGGCTATCAGTGGTCAGAACGCTCAGGCCAAAATGATTGCCGCTCAGACCGCTGCTGGTCGTCGTCAAGCTATGGAAATCATGAGGCAGACGAACATCCAGAATGCTGACCTATCGTTGCAAGCTCGAAGTAAACTTGAGGAAGCGTCCGCCGAGTTGACCCCACAGAAc                                                                                                                    <  1:704176‑M2/187‑1 (MQ=255)`  
                                                                                                                                                                                    `cTCAAGACTATCGGTGCGGTATTCCATGAAGAGTACACACGAGATGGTCAATTTCAGTTATTTACAATCACGAAAGGAGGATAACCATATGTGTTGGGCAGCCGCAATACCTATCGCTATATCTGGCGCTAAGGCTATCAGTGATCAGAACGCTCAGGCCAAAATGATTGCCGCTCAGACCGCTGCTGGTCGTCGTCAAGCTATGGAAATCATGAGGCAGACGAACATCCAGAATGCTGACCTATCGTTGCAAGCTCGAAGTAAACTTGAGGAAGCGTCCGCCGAGTTGACCCCACAGAAc                                                                                                                    <  1:576022/301‑1 (MQ=255)`  
                                                                                                                                                                                        `ggctcggagatgtgtataagagacaggagTACACACGAGATGGTCAATTTCAGTTATTTACAATCACGAAAGGAGGATAACCATATGTGTTGGGCAGCCGCAATACCTATCGCTATATCTGGCGCTCAGGCTATCAGTGATCAGAACGCTCAGGCCAAAATGATTGCCGCTCAGACCGCTGCTGGTCGTCGTCAAGCTATGGAAATCATGAGGCAGACGAACATCCAGAATGCTGACCTATCGTTGCAAGCTCGAAGTAAACTTGAGGAAGCGTCCGCCAAGTTGACCCCACAGAACATGc                                                                                                                <  1:572080/275‑1 (MQ=255)`  
                                                                                                                                                                                        `aGACTATCGGTGCGGTATTCCATGAAGAGTACACACGAGATGGTCAATTTCAGTTATTTACAATCACGAAAGGAGGATAACCATATGTGTTGGGCAGCCGCAATACCTATCGCTATATCTGGCGCTCAGGCTATCAGTGATCAGAACGCTCAGGCCAAAATGATTGCCGCTCAGACCGCTGCTGGTCGTCGTCAAGCTATGGAAATCATGAGGCAGACGAACATCCAGAATGCTGACCTATCGTTGCAAGCTCGAAGTAAACTTGAGGAAGCGTCCGCCGAGTTGACCCCACAGAACATGc                                                                                                                <  1:164551/301‑1 (MQ=255)`  
                                                                                                                                                                                           `cgaTCGGTGCGGTATTCCATGAAGAGTACACACGAGATGGTCAATTTCAGTTATTTACAATCACGAAAGGAGGATAACCATATGTGTTGGGCAGCCGCAATACCTATCGCTATATCTGGCGCTCAGGCTATCAGTGATCAGAACGCTCAGGCCAAAATGATTGCCGCTCAGACCGCTGCTGGTCGTCGTCAAGCTATGGAAATCAAGAGTCAGACGAACATCCAGAATGCTTACCTATCGTTGCAAGCTCGAAGTAAACTTGAGGAAGCGTCCGCCGAGTTGACCCCACAGAACATGCAGa                                                                                                             <  1:425963/299‑1 (MQ=255)`  
                                                                                                                                                                                           `atgtgtataagagacagcATGAAGAGTACACACGAGATGGTCAATTTCAGTTATTTACAATCACGAAAGGAGGATAACCATATGTGTTGGGCAGCCGCAATACCTATCGCTATATCTGGCGCTCAGGCTATCAGTGATCAGAACGCTCAGGCCAAAATGATTGCCGCTCAGACCGCTGCTGGTCGTCGTCAAGCTATGGAAATCATGAGGCAGACGAACATCCAGAATGCTGACCTATCGTTGCAAGCTCGAAGTAAACTTGAGGAAGCGTCCGCCGAGTTGACCACACAGAACATGCAGa                                                                                                             <  1:755258/284‑1 (MQ=255)`  
                                                                                                                                                                                            `attacatgaagagtacacacgagatggtcaatttcagttatttacaatcacgaaaggaggataaccatatgtgttgggcagccgcaatacctatcgctatatctggCGCTATATCTGGCGCTCAGGCTATCAGTGGTCAGAACGCTCAGGCCAAAATGATTGCCGCTCAGACCGCTGCTGGTCGTCGTCAAGCTATGGAAATCATGAGGCAGACGAACATCCAGAATGCTGACCTATCGTTGCAAGCTCGAAGTAAACTTGAGGAAGCGTCCGCCGAGTTGACCTCACAGAACATGCAGaa                                                                                                            <  1:600427‑M2/195‑1 (MQ=255)`  
                                                                                                                                                                                                      `gagtacacacgagatggtcaatttcagttatttacaatcacgaaaggaggataaccatatgtgttgggcagccgcaatacctatcgctatatctggCGCTATATCTGGCGCTCAGGCTATCAGTGGTCAGAACGCTCAGGCCAAAATGATTGCCGCTCAGACCGCTGCTGGTAGTCGTCAAGCTATGGAAATCATGAGGCAGACGAACATCCAGAATGCTGACCTATCGTTGCAAGCTCGAAGTAAACTTGAGGAAGCGTCCGCCGAGTTGACCCCACAGAACATGCAGAAGGTCCAAGCt                                                                                                  <  1:506813‑M2/205‑1 (MQ=255)`  
                                                                                                                                                                                                        `gtacacatgagatggtcaatttcagttatttacaatcacgaaaggaggataaccatatgtgttgggcagccgcaatacctatcgctatatctggCGCTATATCTGGCGCTCAGGCTATCAGTGGTCAGAACGCTCAGGCCAAAATGATTGCCGCTCAGACCGCTGCTGGTCGTCGTCAAGCTATGGAAATCATGAGGCAGACGAACATCCAGAATGCTGACCTATCGTTGCAAGCTCAAAGTAAACTTGAGGAAGCGTCCGCCAAGTCGACCCCCCAGACCATGCAGAAGGTCAAAGCTAt                                                                                                >  1:32483‑M2/95‑301 (MQ=255)`  
                                                                                                                                                                                                        `gtacacacgagatggtcaatttcagttatttacaatcacgaaaggaggataaccatatgtgttgggcagccgcaatacctatcgctatatctggCGCTATATCTGGCGCTCAGGCTATCAGTGGTCAGAACGCTCAGGCCAAAATGATTGCCGCTCAGACCGCTGCTGGTCGTCGTCAAGCTATGGAAATCATGAGGCAGACGAACATCCAGAATGCTGACCTATCGTTGCAAGCTCGAAGTAAACTTGAGGAAgcgtccgccgagttgacccaacagctgtctcttatacacatctccga                                                                                                >  1:116554‑M2/95‑278 (MQ=255)`  
                                                                                                                                                                                                          `tCCATGAATAGTACACACGAGATGGTCAATTTCAGTTATTTACAATCACGAAAGGAGGATAACCATATGTGTTGGGCAGCCGCAATACCTATCGCTATATATGGCGCTCAGGCTATCAGTGATCAGAACGCTCAGGCCAAAATGATTGCCGCTCAGACCGCTGCTGGTCGTCGTCAAGCTATGGAAATCATGAGGCAGACGAACATCCAGAATGCTGACCTATCGTTGCAAGCTCGAAGTAAACTTGAGGAAGCGTCCGCCGAGTTGACCCCACAGAACATGCAGAAGGTCCAAGCTATTg                                                                                              >  1:642301/1‑301 (MQ=255)`  
                                                                                                                                                                                                            `cattaataagacacacCAAATGGTTAATTGCAGTGTTTTTCAATGCGCAAAGGAGGATAACCATATGTGTTGGGCAGCCGCAATACCTATCGCTATATCTTGCGCTCAGGCTATCAGTGATCATAACGCTCAGGCCAAAATGATTGCCGCTCAGACCGCTGCTGGTCGTCGTCAAGCTATGGAAATCATGAGGCAGACGAACATCCAGAATGCTGACCTATCGTTGCAAGCTCGAAGTAAACTTGAGGAAGCGTCCGCCGAGTTGACCCCACAGAACATGCAGAAGGTCCAAGCTATTggg                                                                                            <  1:105305/291‑1 (MQ=255)`  
                                                                                                                                                                                                             `ataagagacagacacGAGATGGTCAATTTCAGTTATTTACAATCACGAAAGGAGGATAACCATATGTGTTTGGCAGCCGCAATACCTATCGCTATATCTGGCGCTCAGGCTATCAGTGATCAGAACGCTCAGGCCAAAATGATTGCCGCTCAGACCGCTGCTGGTCGTCGTCAAGCTATGGAAATCATGAGGCAGACGAACATCCAGAATGCTGACCTATCGTTGCAAGCTCGAAGTAAACTTGAGGAAGCGTCCGCCGAGTTGACCCCACAGAACATGCAGAAGGTCCAAGCTATTGGGt                                                                                           <  1:28158/290‑1 (MQ=255)`  
                                                                                                                                                                                                              `gatgtgtataagagacaggttatttacaatcacgaaaggaggataaccatatgtgttgggcagccgcaatacctatcgctatatctggCGCTATATCTGGCGCTCAGGCTATCAGTGGTCAGAACGCTCAGGCCAAAATGATTGCCGCTCAGACCGCTGCTGGTCGTCGTCAAGCTATGGAAATCATGAGGCAGACGAACATCCAGAATGCTGACCTATCGTTGCAAGCTCGAAGTAAACTTGAGGAAGCGTCCGCCGAGTTGACCTCACAGAACATGCAGAAGGTCCAAGCTATTGGGTc                                                                                          <  1:488514‑M2/213‑1 (MQ=255)`  
                                                                                                                                                                                                              `agacaggTACACACGAGATGGTCAATTTCAGTTATTTACAATCACGAAAGGAGGATAACCATATGTGTTGGGCAGCCGCAATACCTATCGCTATATCTGGCGCTCAGGCTATCAGTGATCAGAACGCTCAGGCCAAAATGATTGCCGCTCAGACCGCTGCTGGTCGTCGTCAAGCTATGGAAATCATGAGGCAGACGAACATCCAGAATGCTGACCTATCGTTGCAAGCTCGAAGTAAACTTGAGGAAGCGTCCGCCGAGTTGACCCCACAGAACATGCAGAAGGTCCAAGCTATTGGGTc                                                                                          <  1:82611/295‑1 (MQ=255)`  
                                                                                                                                                                                                               `gtgtataagagacaggaTGGTCAATTTCAGTTATTTACAATCACGAAAGGAGGATAACCATATGTGTTGGGCAGCCGCAATACCTATCGCTATATCTGGCGCTCAGGCTATCAGTGATCAGAACGCTCAGGCCAAAATGATTGCCGCTCAGACCGCTGCTGGTCGTCGTCAAGCTATGGAAATCATGAGGCAGACGAACATCCAGAATGCTGACCTATCGTTGCAAGCTCGAAGTAAACTTGAGGAAGCGTCCGCCGAGTTGACCCCACAGAACATGCAGAAGGTCCAAGCTATTGGGTCt                                                                                         <  1:142319/286‑1 (MQ=255)`  
                                                                                                                                                                                                               `gAAGAGTACACACGAGATAGTCAATTTCAGTTATTTACAATCACGAAAGGAGGATAACCATATGTGTTGGGCAGCCGCAATACCTATCGCTATATCTGGCGCTCAGGCTATCAGTGATCAGAACGCTCAGGCCAAAATGATTGCCGCTCAGACCGCTGCTGGTCGTCGTCAAGCTATGGAACTCATGAGGCAGACGAACATCAAGAATGCTGACCTATCCTTGCAAGCTCGAATTAAACTTGAGGAAGAGTCCGCCGCGTTGACCCCACAGAAAATCCAGAAGGTCCAAGCTACTTGGTCt                                                                                         >  1:206486/1‑301 (MQ=255)`  
                                                                                                                                                                                                               `aaaGAGTACACACGAGATGGTCAATTTCAGTTATTTACAATCACGAAAGGAGGATAACCATATGTGTTGGGCAGCCGCAATACCTATCGCTATATCTGGCGCTCAGGCTATCAGTGATCAGAACGCTCAGGCCAAAATGATTGCCGCTCAGACCGCTGCTGGTCGTCGTCAAGCTATGGAAATCATGAGGCAGACGAACATCCAGAATGCTGACCTATCGTTGCAAGCTCGAAGTAAACTTGAGGAAGCGTCCGCCGAGTTGACCCCACAGAACATGCAGAAGGTCCAAGCTATTGGGTCt                                                                                         <  1:69986/300‑1 (MQ=255)`  
                                                                                                                                                                                                                 `agagTACACACGAGATGGTCAATTTAAGTTATTTACAATCACGAAAGGAGGATAACCATATGTGTTGGGCAGCCGCAATACCTATCGCTATATCTGGCGCTCAGGCTATCAGTGATCAGAACGCTCAGGCAAAAATGATTGCCGCTCAGACCGCTGCTGGTCGTCGTCAAGGTATGGAAATCATGAGGCAGACGAACATCCAGAATGCTGACCTATCGTTGCAAGCTCGAAGTAAACTTGAGGAAGCGTCCGCCGAGTTGACCACACAGAACATGCAGAAGGTCCAAGCtctgtctcttat                                                                                       >  1:395348/1‑290 (MQ=255)`  
                                                                                                                                                                                                                  `gagTACACACGGGATGGTCAATTTCAGTTAATTACAATCACGAAAGGAGGATAACCATATGTGTTGGGCAGCCGCAATACCTATCGCTATATCTGGAGATCAGGCTATCAGTGATCAGAACGCTCAGGCCAAAATGATTGCCGCTCAGACCGCTGCTGGTCGTCGTCAAGCTATGGAAATCATGAGGCAGACGAACATCCAGAATGCTGACCTATCGTTGCAAGCTCGAAGTAAACTTGAGGAAGCGTACGCCGAGTTGACCCCACAGAACATGCAGAAGGTCCAAGCTATTGGGTCTATc                                                                                      <  1:751415/301‑1 (MQ=255)`  
                                                                                                                                                                                                                  `gagTACACACGAGATGGTCAATTTCAGTTATTTACAATCACGAAAGGAGGATAACCATATGTGTTGGGCAGCCGCAATACCTATCGCTATATCTGGCGCTCAGGCTATCAGTGATCAGAACGCTCAGGCCAAAATGATTGCCGCTCAGACCGCTGCTGGTCGTCGTCAAGCTATGGAAATCATGAGGCAGACGAACATCCAGAATGCTGACCTATCGTTGCAAGCTCGAAGTAAACTTGCTGACGCGTCCGCCGAGTTGACCCCACAGAACCTGCCGAAGGTcctgtctcttatcaacatc                                                                                      >  1:807654/1‑284 (MQ=255)`  
                                                                                                                                                                                                                    `gTACACACGAGATGGTCAATTTCAGTTATTTACAATCACGAAAGGATGATAACCATATGTGTTGGGCAGCCGCAATACCTATCGCTATATCTGGCGCTCAGGCTATCAGTGATCAGAACGCTCAGGCCAAAATGATTGCCGCTCAGACCGCTGCTGGTCGTCGTCAAGCTATGGAAATCATGAGGCAGACGAACATCCAGAATGCTGACCTATCGTTGCAAGCTCGAAGTAAACTTGAGGAAGCGTCCGCCGAGTTGACCCCACAGAACATGCAGAAGGTCCAAGCTATTGGGTCTATCCg                                                                                    <  1:727973/301‑1 (MQ=255)`  
                                                                                                                                                                                                                    `gTACACACGAGATGGTCAATTTCAGTTATTTACAATCACGAAAGGAGGATAACCATATGTGTTTGGCAGCCGCAATACCTATCGCTATATCTGGCGCTCAGGCTATCAGTGATCAGAACGCTCAGTCCAAAATGATTTCCGCTCAGACCGCTGCTTGTCGTCGTCAAGCTATGGAAATCATGAGGCAGACGAACATCCAGAATGCTGACCTATCGTTGCAAGCTCGAAGTAAACTTGAGGAAGCGTCCGCCGAGTTGACCCCACAGAACATGCAGAAGGTACAAGCTATTGGGTCTATCCg                                                                                    >  1:365280/1‑301 (MQ=255)`  
                                                                                                                                                                                                                      `ccacacGAGATGGTCAATTTCAGTTATTTACAATCACGAAAGGAGGATAACCATATGTGTTGGGCAGCCGCAATACCTATCGCTATATCTGGCGCTCAGGCTATCAGTGATCAGAACGCTCAGGCCAAAATGATTGCCGCTCAGACCGCTGCTGGTCGTCGTCAAGCTATGGAAATCATGAGGCAGACGAACATCCAGAATGCTGACCTATCGTTGCAAGCTCGAAGTAAACTTGAGGAAGCGTCCGCCGAGTTGACCCCACAGAACATGCAGAAGGTCCAAGCTATTGGGTCTATCCGAg                                                                                  >  1:267855/2‑301 (MQ=255)`  
                                                                                                                                                                                                                       `ctcggagatgtgtataagagacagaTTTACAATCACGAAAGGAGGATAACCATATGTGTTGGGCAGCCGCAATACCTATCGCTATATCTGGCGCTCAGGCTATCAGTGATCAGAACGCTCAGGCCAAAATGATTGCCGCTCAGACCGCTGCTGGTCGTCGTCAAGCTATGGAAATCATGAGGCAGACGAACATCCAGAATGCTGACCTATCGTTGCAAGCTCGAAGTAAACTTGAGGAAGCGTCCGCCGAGTTGACCTCACAGAACATGCAGAAGGTCCAAGCTATTGGGTCTATCCGAGc                                                                                 <  1:773573/277‑1 (MQ=255)`  
                                                                                                                                                                                                                        `gagatgtgtataagagacagcacgaaaggaggataaccatatgtgttgggcagccgcaatacctatcgctatatctggCGCTATATCTGGCGCTCAGGCTATCAGTGGTCAGAACGCTCAGGCCAAAATGATTGCAGCTCAGACCGCTGCTGGTCGTAGTCAAGCTATGGAAATCATGAGGCAGACGAACATCCAGAATGCTGACCTATCGTTGCAAGCTCGAAGTAAACTTGAGGAAGCGTCCGCCGAGTTGACCCCACAGAACATGCAGAAGGTCCAAGCTATTGGGTCTATCCGAGCg                                                                                <  1:356566‑M2/223‑1 (MQ=255)`  
                                                                                                                                                                                                                        `acacGATATGGTCAATTTCAATTATTTACAATCACGAAAGGAGGATAACCATATGTGTTGGGCAGCCGCAGTACCTATCGCTAGATCTGGCGCTCAGGCTATCAGTGATCAGAACGCTCAGGCCAAAATGATTGCCGCTCAGACCGCTGCTGGTCGTCGTCAAGCTATGGAAATCATGAGGCAGACGAACATCCAGAATGCTGACCTATCGTTGCAAGCTCGAAGTAAACTTGAGGAAGCGTCCGCCGGGTTGACACCACAGAACATGCAGAAGGTCCAAGCTATTGGGTCTATCCGAGCg                                                                                <  1:460166/301‑1 (MQ=255)`  
                                                                                                                                                                                                                                `tGGTCAATTTCAGTTATTTACAATCACGAAAGGAGGATAACCATATGTGTTGGGCAGCCGCAATACCTATCGCTATATCTGGCGCTCAGGCTATCAGTGATCAGAACGCTCAGGCCAAAATGATTGCCGCTCAGACCGCTGCTGGTCGTCGTCAAGCTATGGAAATCATGAGGCAGACGAACATCCAGAATGCTGACCTATCGTTGCAAGCTCGAAGTAAACTTGAGGAAGCGTCCGCCGAGTTGACCCCACAGAACATGCAGAAGGTCCAAGCTATTGGGTCTATCCGAGCGGCTAGCgg                                                                        <  1:253968/301‑1 (MQ=255)`  
                                                                                                                                                                                                                                `gttatttacaatcacgaaaggaggataaccatatgtgttgggcagccgcaatacctatcgctatatctggCGCTATATCTGGCGCTCAGGATATCAGTGGTCAGAACGCTCAGGCCAAAATGATTGCCGCTCAGACCGCTGCTGGTCGTCGTCAAGCTATGGAAATCATGAGGCAGACGAACATCCAGAATGCTGACCTATCGTTGAAAGCTCGAAGTAAACTTGAGGAAGCGTCCGCCGAGTTGACCCCACAGAACATGCAGAAGGTCCAAGCTATTGGGTCTATCCGAGCGGCTATCgg                                                                        <  1:616607‑M2/231‑1 (MQ=255)`  
                                                                                                                                                                                                                                  `gTCAATTTCAGTTATTTACAATCACGAAAGGAGGATAACCATATGTGTTTGGCAGCCGCAATACCTATCGCTATATCTGGCGCTCAGGCTATCAGTGATCAGAACGCTCAGGCCAAAATGATTGCCGCTCAGACCGCTGCTGGTCGTCGTCAAGCTATGGAAATCATGAGGCAGACGAACATCCAGAATGCTGACCTATCGTTGCAAGCTCGAAGTAAACTTGAGGAAGCGTCAGCCGAGTTGACCCCACAGAACATGCAGAAGGTCCAAGCTATTGGGTCTATCCGAGCGGCTATCGgag                                                                      >  1:716087/1‑301 (MQ=255)`  
                                                                                                                                                                                                                                  `aaggagatgtgtataagaaacagtaaccagatgtgttgggcagccgcaatacctatcgctatatctgtCGCTATATCTGGCGCTCAGGCTATCAGTGGTCATAACGCTCAGGCCAAAATGATTGCCGCTCAGACCGCTGCTGGTCGTCGTCAAGCTATGGAAATCATGAGGCAGACGAACATCCAGAATGCTGACCTATCGTTGCAAGCTCTAAGTAAACTTGAGGAAGCGTCCGCCGAGTTGACCTCACAGAACATGCAGAAGGTCCAAGCTATTGGGTCTATCCGAGCGGCTATCGgag                                                                      <  1:264062‑M2/233‑1 (MQ=255)`  
                                                                                                                                                                                                                                    `tttacaatcacgaaaggaggataaccatatgtgttgggcatccgcaatacctatcgctatatctggCGCTATATCTGGCGCTCAGGCTATTAGTGGTCAGAACGCTCAGGCCAAAATGATTGCCGCTCAGACCGCTGCTGGTCGTCGTCAAGCTATGGAAATCATGAGGCAGACGAACATCCAGAATGCTGACCTATCGTTGCAAGCTCGAAGTAAACTTGAGGAAGCGTCCGCCGAGTTGACCCCACAGAACATGCAGAAGGTCCAAGCTATTGGGTCTATCCGAGCGGCTATCGgagag                                                                    <  1:500575‑M2/235‑1 (MQ=255)`  
                                                                                                                                                                                                                                      `aTTTCAGTTATTTACAATCACGAAAGGAGGATAACCATATGTGTTGGGCAGCCGCAATACCTATAGCTATATCTGGAGCTCAGGATATCAGTGATCAGAACGCTCAGGCCAAAATGATTGCCGCTCAGACCGCTGCTGGTCGTCGTCAAGCTATGGAAATCATGAGGCAGACGAACATCCAGAATGCTGACCTATCGTTGCAAGCTCGAAGTAAACTTGAGGAAGCGTCCGCCGAGTTGACCCCACAGAACATGCAGAAGGTCCAAGCTATTGGGTCTATCCGAGCGGCTATCGGagagag                                                                  >  1:621973/1‑301 (MQ=255)`  
                                                                                                                                                                                                                                           `tcacgaaaggaggataaccatatgtgttgggcagccacaatacctatcgctatatctggCGCTATATCTGGCGCTCAGGCTATCAGTGGTCAGAACGCTCAGGCCAAAATGATTGCCGCTCAGACCGCTGCTGGTCGTCGTCAAGCTATGGAAATCATGAGGCAGACGAACATCCAGAATGCTGACCTATCGTTGCAATCTCGAAGTAAACTTGAGGAAGCGTCCGCCGAGTTGACCTCACAGAACATGCAGAAGGTCCAAGCTATTGGGTCTATCCGAGCGGCTATCGGAGAGAGTATGc                                                             >  1:219453‑M2/60‑301 (MQ=255)`  
                                                                                                                                                                                                                                            `gggctcggagatgtgtataagagacagaCCATATGTGTTGGGCAGCCGCAATACCTATCGCTATATCTGGCGCTCAGGCTATCAGTGATCAGAACGCTCAGGCCAAAATGATTGCCGCTCAGACCGCTGCTGGTCGTCGTCAAGCTATGGAAATCATGAGGCAGACGAACATCCATAATGCTGACCTATCGTTGCAAGCTCGAAGTAAACTTGAGGAAGCGTCCGCCGAGTTGACCCCACAGAACATGCAGAAGGTCCAAGCTATTGGGTCTATCCGAGCGGCTATCTGAGAGAGTATGCt                                                            <  1:319650/274‑1 (MQ=255)`  
                                                                                                                                                                                                                                            `gTTACTTACAATCACGAAAGGAGGATAACCATATGTGTTGGGCAGCCGCAATACCTATCGCTATATCTGGCGCTCACGCTATCAGTGATCAGAACGCTCAGGCCAAAATGATTGCCGCTCAGACCGCTGCTGGTCGTCGTCAAGCTATGGAAATCATGAGGCAGACGAACCTCCAGAATGCTGAACTATCGTTGCAAGCTCGAAGTAAACTTGAGGAAGAATCCGCCGAGTTGACCCCACAGAACATGCAGAAGGTCCAAGCTATTGGGTCTATCTGAGCGGCTATCGGAGAGAGTATGCt                                                            <  1:556591/301‑1 (MQ=255)`  
                                                                                                                                                                                                                                            `catgaaaggaggataaacatatgtgttgggcagccgcaatacctatcgctatatctggCGCTATATCTGGCGCTCAGGCTATCAGTGGTCAGAACGCTCAGGCCAAAATGATTGCCGCTAAGACCGATGCTGGTCGTCGTCAAGCTATGGAAATCATGAGGCAGACGAACATCCAGAATGCTGACCTATCGTTGCAAGCTCGAAGTAAACTTGAGGAAGCGTCCGCCGAGTTGACCACACAGAACATGCAGAAGGTCCAAGCTATTGGGTCTATCCGAGCGGCTATCGGAGAGAGTATGCt                                                            <  1:25825‑M2/243‑1 (MQ=255)`  
                                                                                                                                                                                                                                            `agatgtgtataagagacagggaggaTAACCATATGTGTTGGGCAGCCGCAATACCTATCGCTATATCTGGCGCTCAGGCTATCAGTGATCAGAACGCTCAGGCCAAAATGATTGCCGCTCAGACCGCTGCTGGTCGTCGTCAAGCTATGGAAATCATGAGGCAGACGAACATCCAGAATGCTGACCTATCGTTGCAAGCTCGAAGTAAACTTGAGGAAGCGTCCGCCGAGTTGACCCCACAGAACATGCAGAAGGTCCAAGCTATTGGGTCTATCCGAGCGGCTATCGGAGAGAGTATGCt                                                            <  1:329585/282‑1 (MQ=255)`  
                                                                                                                                                                                                                                                   `ggctcggagatgtgtataagagacagtgtgTTGGGCAGCCGCAATACCTATCGCTATATCTGGCGCTCAGGCTATCAGTGATCAGAACGCTCAGGCCAAAATGATTGCCGCTCAGACCGCTGCTGGTCGTCGTCAAGCTATGGAAATCATGAGGCAGACGAACATCCAGAATGCTGACCTATCGTTGCAAGCTCGAAGTAAACTTGAGGAAGCGTCCGCCGAGTTGACCCCACAGAACATGCAGAAGGTCCAAGCTATTGGGTCTATCCGAGCGGCTATAGGAGAGAGTATGCTTGAAGGt                                                     <  1:68209/275‑1 (MQ=255)`  
                                                                                                                                                                                                                                                      `ggataaccatatgtgttgggcagccgcaatacctatcgctatatctggCGCTATATCTGGCGCTCAGGCTATCAGTGGTCAGAACGCTCAGGCCAAAATGATTGCCGCTCAGACCGCTGCTGGTCGTCGTCAAGCTATGGAAATCATGAGGCAGACGAACATCCAGAATGCTGACCTATCGTTGCAAGCTCGAAGTAAACTTGAGGAAGCGTCCGCCGAGTTGACCTCACAGAACATGCAGAAGGTCCAAGCTATTGGgtctatccgagcggctatcggagctgtctcttatacacatctc                                                  >  1:146965‑M2/49‑281 (MQ=255)`  
                                                                                                                                                                                                                                                        `cACGAAAGGAGGATAACCATATGTGTTGGGCAGCCGCAATACCTATCGCTATATCTGGCGCTCAGGCTATCAGTGATCAGAACGCTCAGGCCAAAATGATTGCCGCTCAGACCGCTGCTGGTCGTCGTCAAGCTATGGAAATCATGAGGCAGACGAACATCCAGAATGCTGACCTATCGTTGCAAGCTCGAAGTAAACTTGAGGAAGCGTCCGCCGAGTTGACCCCACAGAACATGCAGAAGGTCCAAGCTATTGGGTCTATCCGAGCGGCTATCGGAGAGAGTATGCTTGAAGGTTCCTc                                                <  1:792381/301‑1 (MQ=255)`  
                                                                                                                                                                                                                                                        `cACGAAAGGAGGATAACCATATGTGTTGGGCAGCCGCAATACCTATCGCTATATCTGGCGCTCAGGATATAAGTGATCAGAACGCTCAGGCCAAAATGATTGCCGCTCAGACCGCTGCTGGTCGTCGTCAAGCTATGGAAATCATGAGGCAGACGAACATCCAGAATGCTGACCTATCGTTGCAAGCTCGAAGTAAACTTGAGGAAGCGTCCGCCGAGTTGAACCCACAGAACATGCAGAAGGTCCAAGCTATTGGGTCTATCCGAGCGGCTATCGGAGAGAGTATGCTTGAAGGTTCCTc                                                >  1:125773/1‑301 (MQ=255)`  
                                                                                                                                                                                                                                                        `cACGAAAGGAGGATAACCATATGTGTTGGGCAGCAGCAATACCTATCGCTATATCTGGCGCTCAGGCTATCAGTGATCAGAACGCTCAGGCCAAAATGATTGCCGCTCAGACCGCTGCTGGTCGTCGTCAAGCTATGGAAATCATGAGGCAGACGAACATCCAGAATGCTGACCTATCGTTGCAAGCTCGAAGTAAACTTGAGGAAGCGTCCACCGAGTTGACCCCACAGAACATGCAGAAGGTCCAAGCTATTGGGTCTATCCGAGCGGCTATCGGAGAGAGTATGCTTGAAGGTTCCTc                                                >  1:310713/1‑301 (MQ=255)`  
                                                                                                                                                                                                                                                                `gaggaTAACCATATGTGTTGGGCAGCCGCAATACCTATCGCTATATCTGGCGCTCAGGCTATCAGTGATCAGAACGCTCAGGCCAAAATGATTGCCGCTCAGACCGCTGCTGGTCGTCGTCAAGCTATGGAAATCATGAGGCAGACGAACATCAAGAATGCTGAACTATCGTTGCAAGCTCGAAGTAAACTTGAGGAAGCGTCCGCCGAGTTGACCCCACAGAACATGCAGAAGGTCCAAGCTATTGGGTCTATCCGAGCGGCTATCGGAGAGAGTATGCTTGAAGGTTCCTCAATGGAcc                                        >  1:441041/1‑301 (MQ=255)`  
                                                                                                                                                                                                                                                                  `gtgttgggcagccgcaatacctatcgctatatctggCGCTATATCTGGCGCTCAGGCTATCAGTGGTCAGAACGCTCAGGCCAAAATGATTGCCGCTCAGACCGCTGCTGGTCGTCGTCAAGCTATGGAAATCATGAGGCAGACGAACATCCATAATGCTGACCTATCGTTGCAAGCTCGAAGTAAACTTGAGGAAGCGTCCGCCGAGTTGACCCCACAGAACATGCAGAAGGTCCaagctattgggtctatccgagcggctatcggagagctgtctcttatacacatctccgagcccacc                                      >  1:51332‑M2/37‑271 (MQ=255)`  
                                                                                                                                                                                                                                                                          `atatGTGTTGGGCAGCCGCAATACCTATCGCTATATCTGGCGCTCAGGCTATCAGTGATCAGAACGCTCAGGCCAAAATGATTTCCGCTCAGACCGCTGCTGGTCGTCGTCAAGCTATGGAAATCATGAGGCAGACGAACTTCCAGAATGCTTACCTATCGTTGCAAGCTCGAAGTAAACTTGAGGAAGCGTCCGCCGAGTTGACCCCACAGAACATGCAGAAGGTCCAAGCTATTGGGTCTATCCGAGCGGCTATCGGAGAGAGTATGCTTGAAGGTTCCTCAATGGACCGCATTAAGCg                              <  1:585526/301‑1 (MQ=255)`  
                                                                                                                                                                                                                                                                          `agacaggtTGGGCAGCCGCAAGACCTATCGCTATATCTGGCGCTTGAGCTATCAGTTATCAGAACGCTCAGGCCAAAATGATTGCCGCTCGGACCGCTGCTGGTCGTAGTCAAGCTATGTAAATCATGAGGCAGACGAACATCCAGAATGCTGACCTATCGTTGCAAGCTCGAAGTAAACTTGAGGAAGCGTCCGCCGAGTTGACCCCACAGAACATGCAGAAGGTCCAAGCTATTTGGTCTATCCGAGAGGCTATCGGAGAGAGTATGCTTGAAGGTTCCTCAATGGACCGCATTAAGCg                              <  1:693048/295‑1 (MQ=255)`  
                                                                                                                                                                                                                                                                              `gtgtTGGGCAGCCGCAATACCTATCGCTATATCTGGCGCTCAGGCTATCAGTGATCAGAACGCTCAGGCCAAAATGATTGCCGCTCAGACCGCTGCTGGTCGTCGTCAAGCTATGGAAATCATGAGGCAGACGAACATCCAGAATGCTGACCTATCGTTGCAAGCTCGAAGTAAACTTGAGGAAGCGTCCGCCGAGTTGACCTCACAGAACATGCAGAAGGTCCAAGCTATTGGGTCTATCCGAGCGGCTATCGGAGAGAGTATGCTTGAAGGTTCCTCAATGGACCGCATTAAGcctgtc                          >  1:594571/1‑296 (MQ=255)`  
                                                                                                                                                                                                                                                                               `gcaatacctatcgctatatctggCTCTATATCTGGCGCTCAGGCTATCAGTGGTCAGAACGCTCAGGCCAAAATGATTGCCGCTCAGACCGCTGCTGGTCGTCGTCAAGCTATGGAAATCATGAGGCAGACGAACATCCAGAATGCTGACCTATCGTTGCAAGCTCGAAGTAAACTTGAGGAAGCGTCCGCCTAGTTGACCCCACAGAACATGCAGAAGGTCCAAGCTATTGGGTCTATCCGAGctgctatcggagagagtatgcttgaaggtctgtctcttatacacatctccgagccca                         >  1:40432‑M2/24‑273 (MQ=255)`  
                                                                                                                                                                                                                                                                                  `ataacgtacgctatatctggAGCTATATCTGGCGCTCAGGCTATCAGTTGTCAGAACGCTCAGGCCAAAATGATTGCAGCTCAGACCGCTGCTGGTCGTCGTCAAGCTATGGAAATCATGAGGCAGACGAACATCCAGAATGCTGACCTATCGTTGCAAGCTCGAAGTAAACTTGAGGAAGCGTCCGCCGAGTTGACCTCAAAGAACATGCAGAAGGTCCAAGCTATTGGGTCTATCCGAGCGGATATCGGAGAGAGTATGCTTGAAGGTTCCTCAATGGACCGCATTAAGCGAGTCACAg                      <  1:317640‑M2/281‑1 (MQ=255)`  
                                                                                                                                                                                                                                                                                        `atcgctatatctggCGCTATATCTGGCGCTCAGGCTATCAGTGGTCAGAACGCTCAGGCCAAAATGATTGCCACTCAGACCGCTGCTGGTCGTCGTCAAGCTATGGAAATCATGAGGCAGACGAACATCCAGAATGCTGACCTATCGTTGCAAGCTCGAAGTAAACTTGAGGAAGCGTCCGCCGAGTTGACCCCACAGAACATGCAGAAGGTCCAAGCTATTGGGTCTATCCGAGCGGCTATCGGAGAGAGTATGCTTGAAGGTTCCTCAATGGACCGCATTAAGCGAGTCACAGAAGGAc                <  1:415589‑M2/287‑1 (MQ=255)`  
                                                                                                                                                                                                                                                                                           `gctatatctggCGCTATATCTGGCGCTCAGGCTATCAGTGGTCAGAACGCTCAGGACAAAATGATTGCCGCTCAGACCGCTGCTGGTCGTCGTCAAGCTATGGAAATCATGAGGCAGACGAACATCCAGAATGCTGACCTATCGTTGCAAGCTCGAAGTAAACTTGAGGAAGCGTCCGCCGAGTTGACCCCACAGAACATGCAGAAGGTCCAAGCTATTGGGTCTATCCGAGCGGCTATCGGAGAGAGTATGCTTGAAGGTTCCTCAATGGACAgcattaagcgagtcctgtctcttatac             >  1:649465‑M2/12‑288 (MQ=255)`  
                                                                                                                                                                                                                                                                                                `aCCTATCGCTATATCTGGCGCTCAGGCTATCAGTGGTCAGAACGCTCAGGCCAAAATGATTGCCGCTCAGACCGCTGCTGGTCGTCGTCAAGCTATGGAAATCATGAGGCAGACGAACATCCAGAATGCTTATCTATCGTTGCAAGCTAGAAGTAAACTTGAGGAAGTGTCCGCCGAGTGGACCTCACAGAACATGCAGAATGGCCAAGCGATTGGTTCTAGCCGACCGGCTATCGGAGAGAGTATGCTTGAAGGTTCCTCAATGGACCGCATTAAGCGAGTCACAGAAGGACAGTTCAtt        >  1:136804/1‑301 (MQ=255)`  
                                                                                                                                                                                                                                                                                                `aCCTATCGCTATATCTGGCGCTCAGGCTATCAGTGATCAGAACGCTCAGTCCAAAATGATTGCCGCTCAGACCGCTGCTGGTCGTCGTCAAGCTATGGAAATCATGAGGCAGACGAACATCCAGAATGCTGACCTATCGTTGCAAGCTCGAAGTAAACTTGAGGAAGCGTCCGCCGAGTTGACCCCACAGAACATGCAGAAGGTCCAAGCTATTGGGTCTATCCGAGCGGCTATCGGAGAGAGTATGCTTGAAGGTTCCTCAATGGACCGCATTAAGCGAGTCACAGAAGGACAGTTCAtt        >  1:199302/1‑301 (MQ=255)`  
                                                                                                                                                                                                                                                                                                `aCCTATCGCTATATCTGGCGCTCAGGCTATCAGTGATCAGAACGCTCAGGCCAAAATGATTGCCGCTCAGACCGCTGCTGGTCGTCGTCAAGCTATGGAAATCATGAGGCATACGAACATCCAGAATGCTGACCTATCGTTGCAAGCTCGAAGTAAACTTGAGGAAGCGTCCGCCGAGTTGACCCCACAGAACATGCAGAAGGTCCAAGCTATTGGGTCTATCCGAGCGGCTATCGGAGAGAGTATGCTTGAAGGTTCCTCAATGGACCACATTAAGcctgtctcttatacacatctccga        >  1:455566/1‑278 (MQ=255)`  
                                                                                                                                                                                                                                                                                                 `ccTATCGCTAGATCTGGCGCTCAGGCTATCAGTGATCAGAACGCTCAGGCCAAAATGATTGCCGCTCAGACCGCTGCTGGTCGTCGTCAAGCTATGGAAATCATGAGGCAGACGAACATCCAGAATGCTGACCTATCGTTGCAAGCTCGAAGTAAACTTGAGGAAGCATCCGCCGAGTTGACCCCACAGAACATGCAGAAGGTCCAAGCTATTGGGTCTATCCGAGCGGCTATCGGAGAGAGTATGCTTGAAGGTTCCTCAATGGACCGCATTAAGCGAGTCACAGAAGGACAGTTCATTc       <  1:191775/301‑1 (MQ=255)`  
                                                                                                                                                                                                                                                                                                      `cGCTATATCTGGCGCTCAGGCTATCAGTGATCAGAACGCTCAGGCCAAAATGATTGCCGCTCAGACCGCTGCTGGTCGTCGTCAAGCTATGGAAATCATGAGGAAGACGAACATCCAGAATGCTGACCTATCGTTGCAAGCTCGAAGTAAACTTGAGGAAGCGTCCGCCGAGTTGACCACACAGAACATGCAGAAGGTCCAAGCTATTGGGTCTATCCGAGCGGCTATCGGAGAGAGTATGCTTGAAGGTTCCTCAATGGACCGCATTAAGCGAGTCACAGAAGGAcctgtctcttataca  >  1:343207/1‑287 (MQ=255)`  

                                                                                                                                                                                                                                                                                                      `|`                                                                                                                                                                                                                                                                                                              
`ATCGGTGGTAACTGCGGGGACCAGTGCTGGTTCGTTACGAGCGACCAAGTGTGGCGACTTAGTGGAAAGGCTAAGCGAAAGTTCCGTAAGTTAATCATGGAGTATCGCGATAAGATGCTTGAGAAGTATGATACTCTTTGGAATTACGTATGGGTAGGCAATACGTCCCACATTCGTTTCCTCAAGACTATCGGTGCGGTATTCCATGAAGAGTACACACGAGATGGTCAATTTCAGTTATTTACAATCACGAAAGGAGGATAACCATATGTGTTGGGCAGCCGCAATACCTATCGCTATATCTGGCGCTCAGGCTATCAGTGGTCAGAACGCTCAGGCCAAAATGATTGCCGCTCAGACCGCTGCTGGTCGTCGTCAAGCTATGGAAATCATGAGGCAGACGAACATCCAGAATGCTGACCTATCGTTGCAAGCTCGAAGTAAACTTGAGGAAGCGTCCGCCGAGTTGACCTCACAGAACATGCAGAAGGTCCAAGCTATTGGGTCTATCCGAGCGGCTATCGGAGAGAGTATGCTTGAAGGTTCCTCAATGGACCGCATTAAGCGAGTCACAGAAGGACAGTTCATTCGGGAA  >  NC_001604.1/27460‑28054`

  

Alignment Legend

Aligned base mismatch/match (shaded by quality score): `ATCG/ATCG` `< 3 ≤` `ATCG/ATCG` `< 11 ≤` `ATCG/ATCG` `< 25 ≤` `ATCG/ATCG` `< 36 ≤` `ATCG/ATCG` `< 38 ≤` `ATCG/ATCG`

Unaligned base: `atcg`    Masked matching base: `atcg`    Alignment gap: `‑`    Deleted base: `‑`

Reads not counted as support for junction

read\_name Not counted due to insufficient overlap past the breakpoint.

read\_name Not counted due to not crossing MOB target site duplication.
